# A Novel Case of Homozygous Interferon Alpha/Beta Receptor Alpha Chain (IFNAR1) Deficiency With Hemophagocytic Lymphohistiocytosis

**DOI:** 10.1093/cid/ciaa1790

**Published:** 2020-11-30

**Authors:** Florian Gothe, Catherine F Hatton, Linh Truong, Zofia Klimova, Veronika Kanderova, Martina Fejtkova, Angela Grainger, Venetia Bigley, Joanna Perthen, Dipayan Mitra, Ales Janda, Eva Fronkova, Dusana Moravcikova, Sophie Hambleton, Christopher J A Duncan

**Affiliations:** 1 Immunity and Inflammation Theme, Translational and Clinical Research Institute, Newcastle University, Newcastle upon Tyne, UK; 2 Department of Pediatrics, Dr. von Hauner Children’s Hospital, University Hospital, Ludwig-Maximilians-Universität Munich, Munich, Germany; 3 Banská Bystrica Children`s University Hospital, Banská Bystrica, Slovakia; 4 Department of Paediatric Haematology and Oncology, 2nd Faculty of Medicine, Charles University and University Hospital Motol, Prague, Czech Republic; 5 Northern Centre for Bone Marrow Transplant, Freeman Hospital, Newcastle upon Tyne Hospitals NHS Foundation Trust, Newcastle upon Tyne, UK; 6 Department of Neuroradiology, Newcastle upon Tyne Hospitals NHS Foundation Trust, Newcastle upon Tyne, UK; 7 Department of Pediatrics and Adolescent Medicine, University Medical Center Ulm, Germany; 8 Children’s Immunology Service, Great North Children’s Hospital, Newcastle upon Tyne Hospitals NHS Foundation Trust, Newcastle upon Tyne, UK; 9 Infection and Tropical Medicine, Royal Victoria Infirmary, Newcastle upon Tyne Hospitals NHS Foundation Trust, Newcastle upon Tyne, UK

**Keywords:** IFNAR1, type I interferon, HLH, inborn error of immunity

## Abstract

We present a case of complete deficiency of the interferon alpha/beta receptor alpha chain (IFNAR1) in a child with fatal systemic hyperinflammation, apparently provoked by live-attenuated viral vaccination. Such pathologic hyperinflammation, fulfilling criteria for hemophagocytic lymphohistiocytosis, is an emerging phenotype accompanying inborn errors of type I interferon immunity.

**(See the Editorial Commentary by Mogensen on pages 140–3.)**

Type I interferons (including IFN-β and 13 IFN-α subtypes) signal via a single ubiquitously expressed receptor composed of low- and high-affinity subunits, IFNAR1 and IFNAR2. Type I IFNs appear critical to mammalian antiviral immunity, based on extensive studies of *Ifnar1-*deficient mice, but their precise role in humans was hitherto uncertain [1]. Our discovery of complete human IFNAR2 deficiency in a child with fatal viral disease secondary to live-attenuated measles mumps and rubella (MMR) vaccination implied an essential antiviral function [2]. It was nevertheless striking that the proband displayed no overt viral susceptibility prior to MMR exposure. This phenotype of disease provoked by challenge with systemic live-attenuated viral vaccination was seen in 2 patients with IFNAR1 deficiency, both similarly healthy until vaccination [3]. However, the recent identification of *IFNAR1* variants in 2 patients with severe acute respiratory syndrome coronavirus 2 (SARS-CoV-2) highlights the additional importance of type I IFNs in immunity to naturally acquired viruses [4].

In this study, we investigated a previously well 15-month-old boy who developed severe illness following MMR vaccination, comprising progressive and ultimately fatal hyperinflammation meeting diagnostic criteria for hemophagocytic lymphohistiocytosis (HLH; see case summary, Figure 1A, Supplementary Table E1). Analysis of serum (Supplementary Table E2) and cerebrospinal fluid (Supplementary Table E3) did not yield evidence of vaccine-strain viral replication, nor was an alternative infectious etiology identified, other than low-level Epstein-Barr virus (EBV) reactivation. Detailed immune phenotyping showed monocytosis and T-cell lymphocytosis, with low numbers of B cells and cDC1 (Supplementary Figure E1, Supplementary Table E4). Despite aggressive antiviral, immunomodulatory therapy, and intravenous immunoglobulins (IVIG), he developed encephalopathy with abnormal neuroimaging and succumbed suddenly approximately 6 months after presentation (Supplementary Figure E2–E4). Rapid diagnostic whole exome sequencing revealed a novel homozygous nonsense variant (c.922C>T) in *IFNAR1*, introducing a premature stop codon in the third extracellular domain of IFNAR1 protein (p.Gln308Ter, Figure 1B). This variant was absent from the GnomAD database and predicted to be deleterious by in silico tools (Supplementary Table E5). No protein product was detected when probing primary dermal fibroblast lysates with N-terminal (Figure 1C) or C-terminal IFNAR1 antibody (Supplementary Figure E5), indicative of complete IFNAR1 deficiency. Scrutiny of a comprehensive panel of genes linked to inborn errors of immunity within the patient’s exome data revealed no additional candidate disease-causing variants.

**Figure 1.**
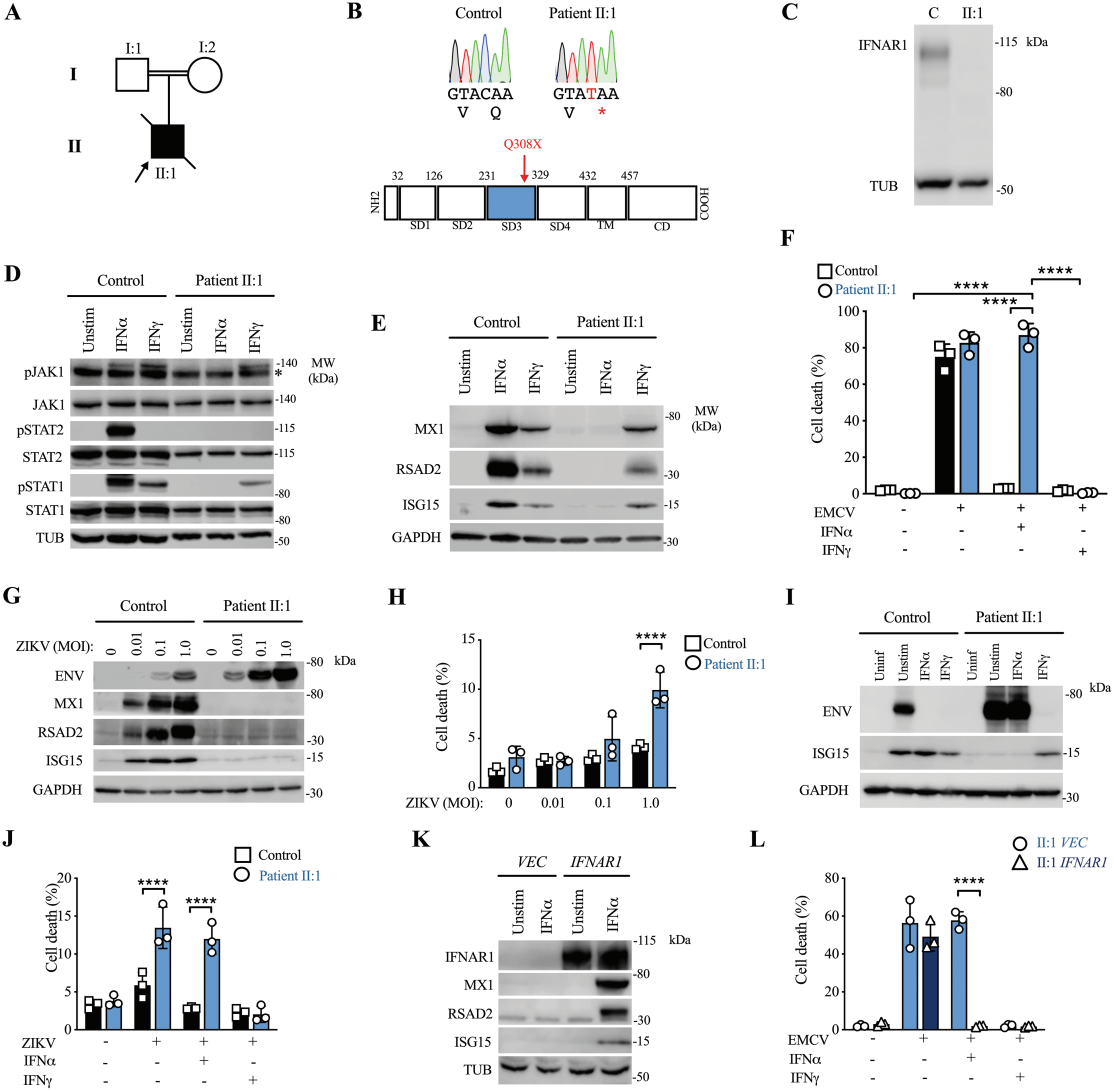
*A*, Pedigree. *B*, Sanger sequencing confirmation of variant with IFNAR1 protein domains. *C*, IFNAR1 deficiency (immunoblot). *D*, Signaling by IFN-α2b or IFN-γ (1000 IU/mL, 30 m, immunoblot). *E*, ISG induction by IFN-α2b or IFN-γ (1000 IU/mL, 16 h, immunoblot). *F*, EMCV cytopathicity protection assay. *G*, ZIKV envelope (ENV), ISG15, RSAD2, and MX1 expression (immunoblot) in fibroblasts (*H*) ZIKV cytopathicity assay. Protection against ZIKV by IFN-α2b or IFN-γ (MOI 1.0, 1000 IU/mL, 24 h) by (*I*) immunoblot and (*J*) viability assay. Complementation with *IFNAR1* but not empty vector (*VEC*) restores (*K*) ISG induction by IFN-α2b (1000 IU/mL, 16 h, immunoblot) and (*L*) IFN-α2b-mediated protection against EMCV. All experiments repeated n = 3 times in II:1 and control primary fibroblasts. Mean ± SD. ****P < .001, 2-way ANOVA with Tukey’s post-test. *nonspecific band. Abbreviations: ANOVA, analysis of variance; EMCV, encephalomyocarditis virus; IFN, interferon; IFNAR1, interferon alpha/beta receptor alpha chain; ISG, interferon-stimulated gene; MOI, multiplicity of infection; ZIKV, Zika virus.

Upon ligand binding, the ternary complex of IFNAR1-IFN-IFNAR2 initiates an intracellular signaling cascade in which reciprocal transphosphorylation of the receptor-associated kinases JAK1 and TYK2 is followed by phosphorylation of the signal transducers and activators of transcription STAT1 and STAT2. The majority of the transcriptional response to IFN-αβ is attributable to the heterotrimer formed of phosphorylated STAT1 and STAT2 together with IRF9. This complex, known as interferon stimulated gene factor 3 (ISGF3), translocates to the nucleus where it interacts with interferon-sensitive response elements (ISREs) to activate the transcription of a large number of interferon-stimulated genes (ISGs). A fraction of the phospho-STAT1 instead homodimerizes to form the IFN-γ activation factor (GAF), agonizing a distinct but overlapping set of genes bearing IFN-γ activation sites (GAS), more typically associated with type II IFN (IFN-γ) signaling. The predicted effect of IFNAR1 deficiency is to prevent signaling and downstream functional responses to IFN-αβ but leave intact responses to IFN-γ.

Consistent with this prediction, phosphorylation of JAK1 and its downstream targets STAT1 and STAT2 was undetectable in IFNAR1-deficient patient fibroblasts exposed to IFN-α2b for 30 minutes, as revealed by immunoblotting of whole cell lysates, whereas phosphorylation of JAK1 and STAT1 upon exposure to IFN-γ was preserved (Figure 1D). The same pattern was observed by analysis of STAT1 phosphorylation in patient lymphocytes and monocytes by phospho-flow cytometry (Supplementary Figure E6). Accordingly, immunoblotting revealed failure to induce ISG protein products in IFNAR1-deficient patient fibroblasts exposed overnight to IFN-α2b despite intact responses to IFN-γ (Figure 1E). To address the functional impact, we challenged cells with the picornavirus encephalomyocarditis virus (EMCV). In this experiment, fibroblasts were pretreated with IFN-α2b or IFN-γ overnight prior to infection, at a dose that was previously determined to prevent cytopathic effect (CPE) in control cells, and then examined at 24 hours postinfection. IFNAR1-deficient cells were susceptible to CPE despite IFN-α2b exposure, but were rescued by IFN-γ treatment, confirming a specific defect of IFN-αβ-mediated antiviral immunity (Figure 1F).

To extend and confirm these findings, we infected cells with the flavivirus Zika virus (ZIKV) at different multiplicities of infection, examining expression of viral envelope protein and ISGs by immunoblot at 48 hours postinfection (Figure 1G). In patient cells, we saw excessive viral protein expression (Supplementary Figure E7), accompanied by the failure to induce expression of ISG15, RSAD2, and MX1, reflecting a defect of IFNAR-mediated antiviral resistance and correlated with susceptibility to viral cytotoxicity (Figure 1H). Treatment of patient cells with exogenous IFN-γ but not IFN-α2b prevented ZIKV infection (Figure 1I) and CPE (Figure 1J).

To prove definitively that the loss of IFNAR1 was responsible, we complemented patient fibroblast cells with full-length human *IFNAR1* delivered by lentiviral transduction. Lentiviral transduction of *IFNAR1* in patient fibroblasts, but not empty vector, restored induction of ISGs (Figure 1K) and formation of an antiviral state in response to IFN-α2b (Figure 1L), thereby confirming the genotype-phenotype association.

We report a new case of complete IFNAR1 deficiency, complementing a recent report of homozygous IFNAR1 deficiency in 2 unrelated children [3]. In all 3 of these cases and similar to IFNAR2-deficient patients [2], viral susceptibility became apparent only following inoculation with live-attenuated viral vaccines. At first sight, this contrasts with the broader susceptibility of STAT2- or IRF9-deficient patients [5–7] to viruses naturally encountered at mucosal surfaces, which might be explicable in terms of functional redundancy between type I and type III IFNs at the mucosa [1]. However, the recent discovery of 2 further cases of IFNAR1 deficiency among persons with severe coronavirus disease 2019 (COVID-19) implies that, for SARS-CoV-2 at least, type I IFNs can play a defining role in the host response to natural pathogens [4]. This conclusion is further supported by the detection of neutralizing antibodies against type I but not type III IFNs in 10% of severe COVID-19 cases [4], as well as a genome-wide association signal with *IFNAR2* in an independent cohort [8].

Our case expands the phenotypic spectrum of IFNAR1 deficiency to include HLH-like hyperinflammation, previously noted in IFNAR2 deficiency and increasingly recognized in other defects of IFN-αβ immunity such as STAT2 or IRF9 defects [7, 9]. Defining the pathomechanism of hyperinflammation in this context, particularly its relationship to dysregulated inflammatory signaling and/or viral replication, is an important area for future work. Despite the temporal association with vaccination, our case was notable for the absence of detectable vaccine-strain dissemination. A similar picture has been observed in some STAT1- and STAT2-deficient patients with hyperinflammation [12, 11]. Although we could not exclude a contribution of low-level EBV reactivation, sterile autoinflammation has emerged as a distinct clinical phenotype in defects of IFN-αβ signaling [7]. This suggests a more complex mechanism involving the loss of IFN-αβ regulation [1]. On the face of it this is paradoxical, because uncontrolled IFN-αβ signaling is itself associated with HLH-like sterile inflammation [12]. Yet IFN-αβ also negatively regulates various cytokine pathways, including interleukin (IL)-1β [13] and IL-17 [14]. Immunomodulatory properties of IFN-β are exploited in therapy for immune-mediated diseases such as multiple sclerosis. The partial clinical response to corticosteroids and IL-1β blockade in our case lends some support to the hypothesis that loss of IFN-αβ signaling contributes to disordered immune network activity. In the context of viral disease, it appears that a correctly calibrated IFN-αβ response is necessary to prevent immunopathology. This is the “goldilocks” principle of immune homeostasis, where both inadequate and excessive activity contributes to disease.

Hyperinflammation is central to the pathogenesis of severe COVID-19, as demonstrated by the clinical effectiveness of corticosteroids. Our novel case of IFNAR1 deficiency reveals a potential role for IFN-αβ in preventing hyperinflammation, not simply through direct control of viral replication [2, 3] but possibly also via its immunoregulatory action toward other cytokines. The loss of both aspects of IFN-αβ function might contribute to the susceptibility of patients with *IFNAR1* defects to severe COVID-19 [4, 9]. Understanding the cellular and molecular links between defective IFN-αβ signaling and pathogenic inflammation will be critical in guiding therapy for severe viral disease.

## Supplementary Data

Supplementary materials are available at *Clinical Infectious Diseases* online. Consisting of data provided by the authors to benefit the reader, the posted materials are not copyedited and are the sole responsibility of the authors, so questions or comments should be addressed to the corresponding author.

ciaa1790_suppl_Supplementary_Materials

## Note

***Potential conflicts of interest.*** F. G. received funding from the Deutsche Forschungsgemeinschaft (GO2955/1-1) as well as the Bubble Foundation. C. F. H. is funded by a Medical Research Council (MRC) studentship (MR/N013840/1). V. K., E. F., and M. F. are funded by the Ministry of Health of the Czech Republic (NV19-05-00332). S. H. and C. J. A. D. are funded by the Wellcome Trust (207556/Z/17/Z and 211153/Z/18/Z, respectively). All other authors have no potential conflicts. All authors have submitted the ICMJE Form for Disclosure of Potential Conflicts of Interest. Conflicts that the editors consider relevant to the content of the manuscript have been disclosed.
