# Supplementary material for: A Novel Case of Homozygous Interferon Alpha/Beta Receptor Alpha Chain (IFNAR1) Deficiency With Hemophagocytic Lymphohistiocytosis
Source: Clin Infect Dis. 2020 Nov 30;74(1):136–9. doi: 10.1093/cid/ciaa1790 (PMC8752251; doi:10.1093/cid/ciaa1790)
Supplement: ciaa1790_suppl_Supplementary_Materials [file ciaa1790_suppl_supplementary_materials.docx]

**Supplementary Methods**

**Ethics statement and consent**

This study was performed in accordance with the principles of the Helsinki declaration. Parental consent was obtained for genetic testing. Ethical approval for studies on patient fibroblasts was granted by the NRES Committee North East - Newcastle & North Tyneside 1 (Ref: 16/NE/0002).

**Whole exome sequencing**

Whole exome sequencing of DNA extracted from whole blood of the proband and downstream variant analysis was performed as previously described (1). Sanger sequencing to confirm the variant was undertaken according to standard methods (primer sequences available on request). Parental DNA was not available for segregation studies.

***In silico* prediction tools**

PhastCons is a method to determine the grade of conservation of a given nucleotide, given as a score from 0-1 (2). MutationTaster uses values which are precomputed and offered by UCSC (3). The Combined Annotation Dependent Depletion (CADD) score is a tool for integrating conservation and deleteriousness predictions (4).

**Cells, cytokines, immunoblotting**

Dermal fibroblasts from patient II:1 and three healthy controls were obtained by standard methods and cultured in Dulbecco’s Modified Eagle’s Medium supplemented by 10% fetal calf serum and 1% penicillin/streptomycin (DMEM-10). Human recombinant IFNα2b (Intron-A, Schering-Plough, USA) and IFNγ (Immunikin, Boehringer Ingelheim, Germany) were used at 1000 IU/mL. Immunoblotting was carried out as previously described (5).

| **Antibody** | **Host** | **Dilution** | **Source** | **Code** |
| --- | --- | --- | --- | --- |
| IFNAR1 (N-terminal) | Rabbit | 1:200 | Abcam | ab124764 |
| IFNAR1 (C-terminal) | Rabbit | 1:200 | Abcam | ab45172 |
| RSAD2 | Rabbit | 1:1000 | CST | 13996 |
| ISG15 | Rabbit | 1:1000 | CST | 2743 |
| STAT2 | Mouse | 1:2000 | SCB | sc-1668 |
| pSTAT2 | Rabbit | 1:2000 | CST | 8841 |
| STAT1 | Rabbit | 1:1000 | CST | 9172 |
| pSTAT1 | Rabbit | 1:1000 | CST | 7649 |
| JAK1 | Rabbit | 1:500 | CST | 3344 |
| pJAK1 | Rabbit | 1:500 | CST | 74129 |
| MxA | Rabbit | 1:1000 | SCB | sc-50509 |
| Zika Envelope | Mouse | 1:5000 | BioFront Tech | BF-1176-56 |
| α-tubulin | Mouse | 1:10,000 | CST | 3873 |
| GAPDH | Rabbit | 1:10,000 | CST | 5174 |
| Anti-rabbit HRP- conjugated | Goat | Various | CST | 7074 |
| Anti-mouse HRP-conjugated | Horse | Various | CST | 7076 |

CST = Cell Signalling Technologies; SCB = Santa-Cruz Biotechnology.

**Trucount analysis**

Peripheral blood absolute cell counts were obtained using TruCount™ tubes (BD Biosciences) with 200μl whole blood, stained with a surface antibody cocktail for 30 mins at room temperature before dilution in 900μl red cell lysis buffer. Analysis was performed with an LSRFortessa X-20 (BD Biosciences) running BD FACSDIVA™ 8.0.1 software and data processed with FlowJo 10.6.2 (Tree Star, Inc.) and Graphpad Prism 8.4.1.

| **Antigen** | **Fluorochrome** | **Clone** | **Manufacturer** |
| --- | --- | --- | --- |
| BTLA | PECF594 | J168-540 | BD |
| CD123 | BUV395 | 7G3 | BD |
| CD14 | BV650 | M5E2 | Biolegend |
| CD16 | BV510 | 3G8 | BD/Biolegend |
| CD163 | BV711 | GHI/61 | Biolegend |
| CD19 | PERCPCy5.5 | SJ25C1 | BD |
| CD1c | PE-Cy7 | L161 | Biolegend |
| CD2 | BV421 | RPA-2.10 | Biolegend |
| CD3 | FITC/AF700 | SK7(Leu4) | BD/Biolegend |
| CD303 | BV605 | 201A | Biolegend |
| CD304 | BV605 | 12C2/U21-1283 | Biolegend/BD |
| CD34 | APCCy7 | 581 | Biolegend |
| CD4 | PE | SK3 (Leu3a) | BD |
| CD45 | AF700 | HI30 | Biolegend |
| CD5 | BUV730 | UCHT2 | BD |
| CD56 | APC | NCAM16.2 | BD |
| CD8 | APC-Cy7 | SK1 | BD |
| CD88 | PERCPCy5.5 | S5/1 | Biolegend |
| Clec9A | APC | 8F9 | Biolegend |
| HLA-DR | BV785 | L243 | Biolegend |

BD = BD Biosciences

**Phosflow**

Peripheral blood mononuclear cells (PBMC) from patient and healthy transport control were stimulated with IFNα (0.5 μg/ml, Abcam, Cambridge, UK) or IFNγ (0.5 μg/ml, R&D Systems, Minneapolis, MN) for 15 or 30 min at 37°C and intracellular signaling was arrested using 4% formaldehyde for 10 min at room temperature. Erythrocytes were lysed using 0.1% Triton X-100 for 15 min at 37°C and the leukocytes were permeabilized using 80% ice-cold methanol. B- and T-lymphocytes and monocytes were discriminated according to CD45-APC-H7 (BD Biosciences, San Jose, CA), CD19-PE-Cy7 (Beckman Coulter, Miami, FL), CD3-PerCP-Cy5.5 (Exbio, Vestec, Czech Republic) and CD14-Brilliant Violet 605 (Biolegend, San Diego, CA), and phosphorylated STAT1 (Tyr701) (BD Biosciences). Data were acquired with LSRII flow cytometer and analyzed with FlowJo software (BD Biosciences).

**Virus infections**

Primary human dermal fibroblasts were infected with encephalomyocarditis virus (kindly provided by D. Young and R. Randall, St Andrew’s University, UK) at 10^5^ pfu/mL for 24 hours or with Zika virus strain H/FP/2013 (kindly provided by W. James, University of Oxford, UK) at multiplicity of infection (MOI) = 1.0 (for IFN treatment experiments) or MOI = 1.0 to 0.01. At 2 hours post infection, the inoculum was removed and replaced with fresh medium (DMEM-10), with or without IFN treatment as indicated, for 48 hours prior to imaging analysis or lysis with RIPA buffer (150 mM sodium chloride, 50 mM Tris pH 8, 1% triton X-100, 0.5% sodium dodecyl sulphate) supplemented with phosphatase inhibitor (Roche), 1mM sodium orthovanadate, 10mM sodium fluoride, 10% dithiothreitol (Sigma-Aldrich) and 25% NuPAGE LDS sample buffer (ThermoFisher).

**Cell viability assay**

After dermal fibroblast monolayers were treated as stated, the medium was removed and replaced with live cell imaging solution (ThermoFisher) containing 2 drops per mL of propidium iodide and Hoechst. Plates were then incubated for 15 minutes at 37°C and 5% CO_2_ before being imaged using an EVOS FL fluorescence microscope (ThermoFisher). Image analysis was performed using a bespoke pipeline developed in CellProfiler (Broad institute) to calculate the percentage of dead cells in each condition (Hanrath et al, manuscript in preparation). All experiments were performed in technical duplicate and the average value of n=4 images per well was used for analysis. All experiments were performed at least three times.

**Lentiviral cloning, viral production and complementation**

The destination vector IFNAR1_pCSdest was a kind gift of R. Reeves (Johns Hopkins University, Baltimore, USA). The *IFNAR1* insert was cloned into the Gateway® pDONR207 entry vector (ThermoFisher) using BP clonase (ThermoFisher) and its sequence was confirmed by Sanger-sequencing. It was further inserted into the pLenti-EF1a-GATEWAY-RSV-Puromycin-GFP (AMS Biotechnology, UK) vector using LR clonase (ThermoFisher). Lentiviruses were produced by co-transfection of psPAX2, pCMV-VSV-G and lentiviral transfer plasmid in HEK293FT cells (ThermoFisher) using polyethylenimine (Sigma-Aldrich, Gillingham, UK). Virus-containing supernatants were harvested at 48 h post-transfection, filtered (0.45 μm sterile filter) and concentrated 100-fold with Lenti-X™ Concentrator (TaKaRa, Shiga, Japan) according to manufacturer’s instructions. Cells were spinoculated in 6-well plates (1.5 h, 2000 rpm), with target or null control viral particles in a total volume of 0.5 mL DMEM-10 containing hexadimethrine bromide (Polybrene, 6 mg/mL, Sigma-Aldrich). Cells were rested in virus-containing medium for 4 hrs then incubated in fresh DMEM-10 until 48 h, when they were subjected to selection with 0.75 mg/mL puromycin (Gibco). Antibiotic-containing medium was refreshed every 72 h.

**Statistical analysis**

All experiments were repeated a minimum of n=3 times with multiple control lines. The mean of technical replicates for each experiment was used for data analysis. Data were analysed using Prism version 8.0 (GraphPad Software, San Diego, USA) by two-way ANOVA with Tukey’s post-test correction for multiple comparisons. Adjusted alpha < 0.05 was considered statistically significant.

**Case Summary**

The patient, a 15-month-old boy of consanguineous parentage, presented with fever up to 40°C and transient exanthem five days after the first dose of measles, mumps, rubella (MMR) vaccination. He had been born at term, had no past history other than mild laryngeal stenosis and had received prior routine childhood vaccinations without incident (not including prior live-viral vaccines). Family history revealed two unexplained deaths of infants in his grandparent’s generation. Examination revealed tonsillitis and clindamycin was initiated. A pharyngeal swab taken at this first presentation was positive for Epstein-Barr virus (EBV) by PCR and metizoprinol/isoprinosine (100 mg/kg/day) was added. Over the following ten days the boy suffered from recurrent fever and deterioration of his clinical condition resulted in hospital referral. On admission, generalised lymphadenopathy, hepatosplenomegaly, oedema and a salmon pink exanthem on the trunk were noted. Radiologic evaluation revealed polyserositis with cardiac and pleural effusion as well as ascites. Initial laboratory results showed increased C-reactive protein (94 mg/L) and interleukin-6 (77.9 ng/L) levels as well as thrombocytopenia. Additional laboratory abnormalities, including low fibrinogen, elevated triglycerides as well as high ferritin and elevated soluble CD25, supported the diagnosis of haemophagocytic lymphohistiocytosis (HLH) (Table E1). Bone marrow aspirate revealed macrophage activation but no haemophagocytosis. Investigation for bacterial and viral infection in CSF (Table E3) and blood (Table E4), including PCR testing for CMV, HSV-1/2 and VZV, borrelia, measles, mumps and rubella, was negative. Serology showed IgM against Parvo B19, of uncertain significance, and IgG was reactive against EBV (IgM not detected), consistent with previous exposure to EBV. PCR testing revealed low-level EBV positivity in blood (283 IU/mL) but was otherwise negative. Additional IgG against HSV, VZV and CMV without corresponding IgM positivity was noted, whereas baseline serology was negative for HIV, HBV, HCV, Toxocara and Toxoplasma. He was treated with antivirals (aciclovir 250 mg/m^2^) and for presumed HLH with corticosteroids, cyclosporin A and anakinra (10 mg/kg/day) with additional IVIG (0.4 mg/kg/day). Substantial clinical improvement was seen within two days, with resolution of fever and substantial improvement in laboratory results (Figure E4). Genetic testing for variants in primary HLH genes was negative.

However, fever recurred a few days later although he was not at this point unwell. Lymphopenia and monocytosis were noted alongside kidney injury, accompanying high serum-levels of cyclosporin A, which was discontinued (day 15). Screening for infectious agents in blood was again indicative of low-level EBV reactivation (PCR 1773 IU/mL). Despite treatment escalation with famaciclovir (12.5 mg/kg/day) in addition to acyclovir (500 mg/m^2^) and IVIG (0.4 mg/kg/day), the patient developed a progressive systemic inflammatory response syndrome with respiratory failure. Mechanical ventilation was necessary for five days before a stepwise improvement of the boy’s condition was achieved over several weeks, during which time prednisolone and anakinra were weaned down. At this point, genetic testing by whole exome sequencing was undertaken, identifying a nonsense mutation in *IFNAR1*. The boy was discharged on antibacterial, antiviral and antifungal prophylaxis, regular IVIG administrations were commenced and prednisolone and anakinra were subsequently stopped (day 53).

Two weeks later the patient was readmitted with fever, generalized seizures, hyponatraemia and treatment-refractory hypertension (day 67). Screening for viral disease in blood (day 77) and cerebrospinal fluid (day 81) was again negative for viruses including measles, mumps and rubella although EBV viremia had still been detected a week earlier (PCR 3480 IU/mL at day 70). Cranial MRI revealed symmetric, bilaterally distributed high T2 signal within the parieto-occipital white matter (Figure E2A-D). Additionally, a diffusion restriction in the left hippocampus (Figure E2E) and a generalized cerebral atrophy were noted. On the same day, elevated CSF protein (950 mg/L) was detected. On follow-up scanning six weeks later (day 122), when the patient was still ventilation-dependent and unconscious, the posterior white matter signal changes had resolved (Figure E3). However, the cerebral atrophy worsened between the scans, with further generalized loss of volume specifically in the left medial temporal lobe. Whereas the posterior white matter changes and their resolution over time alone raise the possibility of posterior reversible encephalopathy syndrome (PRES), the involvement of the left hippocampus advocates for an inflammatory or infective aetiology. The dose of IVIG was increased (2 g/kg/day). However, limited neurological improvement was observed and he was transferred at the request of his parents to the local hospital, where he sadly died of sudden cardiorespiratory failure at the age of 21 months.

The aetiology of HLH in this case was unclear. There was clearly a temporal association with MMR administration, however the absence of detection of MMR viruses in CSF or serum argued against MMR dissemination as the cause. EBV reactivation may have been a factor in driving HLH, however this remained at relatively low levels. It is also possible that an additional unidentified infection may have contributed. However, in retrospect, the correlation between clinical disease activity and inflammatory markers (including CRP and IL6), and the initial response to corticosteroid and anakinra, suggested an inflammatory component.

| **2004 Criteria** | **Patient II:1** |
| --- | --- |
| Clinical criteria | |
| Fever | Yes |
| Splenomegaly | Yes |
| Laboratory criteria | |
| Cytopenia | Yes |
| Fibrinogen < 1.5g/L | Yes (0.9 g/L) |
| Triglycerides > 265 mg/dL | Yes (331.8 mg/dL) |
| Ferritin > 500 ng/mL | Yes (1840 ng/mL) |
| sCD25 > 2400 U/mL | Yes (5840 U/mL) |
| Histopathological criteria | |
| Haemophagocytosis | No |

**Table E1**. HLH diagnostic criteria according to HLH-2004 guidelines. A score of ≥ 5/8 supports HLH.

|  | Day -8 | Day 6 | Day 19 | Day 28 | Day 46 | Day 70 | Day 77 |
| --- | --- | --- | --- | --- | --- | --- | --- |
| EBV | IgM -  IgG +  PCR - | PCR +  283 IU/mL | PCR + 1773 IU/mL | PCR + 4347 IU/mL | PCR +  551 IU/mL | PCR + 3480 IU/mL | IgM -  IgG +  PCR - |
| HSV | IgM -  IgG +  PCR- |  |  |  |  |  | IgM -  IgG +  PCR - |
| VZV | IgM -  IgG +  PCR - |  |  |  |  |  | IgM -  IgG +  PCR - |
| CMV | IgM -  IgG +  PCR - |  |  |  |  |  | IgM -  IgG +  PCR - |
| Parvo B19 | IgM +  IgG - |  |  |  | IgM +  IgG - |  |  |
| Measles | IgM -  IgG -  PCR - |  |  |  |  |  | IgM +  IgG +  PCR - |
| Mumps | IgM -  IgG -  PCR - |  |  |  |  |  | IgM +  IgG -  PCR - |
| Rubella | IgM -  IgG -  PCR - |  |  |  |  |  | IgM -  IgG -  PCR - |

**Table E2**. Virologic assessment in blood. Day -8 refers to tests performed in an outpatient setting prior to hospital admission.

|  | Normal range | Day 6 | Day 81 | Day 131 |
| --- | --- | --- | --- | --- |
| Cell count | Less than 15 | 3 (lymphocytes) | 10 (mostly lymphocytes) | 6 (lymphocytes) |
| Glucose [mmol/L] | 2.47-4.12 | 3.59 | 4.25 | 4.05 |
| Lactate [mmol/L] | 1.1-2.8 | 1.12 | 1.16 | 1.12 |
| Protein [mg/L] | 150-450 | 180 | 950 | 567 |
| Multiplex PCR [HSV, VZV, EBV, CMV, measles, rubella and mumps] | Negative | Negative | Negative | Negative |
| Antibodies [Borrelia] | Negative | Negative | Negative | Negative |

**Table E3.** Analysis of cerebrospinal fluid. Multiplex PCR and serology tested for HSV, VZV, EBV, CMV, borrelia, measles, rubella and mumps.

| **Parameter** | **Value** | **Normal range** |
| --- | --- | --- |
| Total lymphocytes | 1030 | 1600-6700 |
| CD3+ CD4+ | 5080 | 1000-4600 |
| CD3+ CD8+ | 1670 | 400-2100 |
| CD3- CD19+ | 2690 | 600-2700 |
| CD3- CD56+ | 240 | 200-1200 |
| IgG (g/L) | 4.03 | 2.75-10 |
| IgM (g/L) | 3.39 | 0.85-1.18 |
| IgA (g/L) | 0.43 | 0.1-0.77 |
| IgE (IU/mL) | 23.2 | <60 |
| Tetanus (mg/L) | 0.21 mg/L | >0.15 |
| Diphteria (mg/L) | 0.11 mg/L | >0.1 |
| Haemophilus (IU/mL) | 1.14 IU/mL | >1.0 |
| Pneumoccocus (IU/mL) | 9.97 IU/mL | >20 |

**Table E4**. Diagnostic investigation results.

| **PhastCons** | **MutTaster** | **CADD** |
| --- | --- | --- |
| 0.57 (0-1) | Disease-causing | 35 (Max. 36) |

**Table E5.** In silico predictions of deleteriousness of c.922C>T *IFNAR1* variant.

**
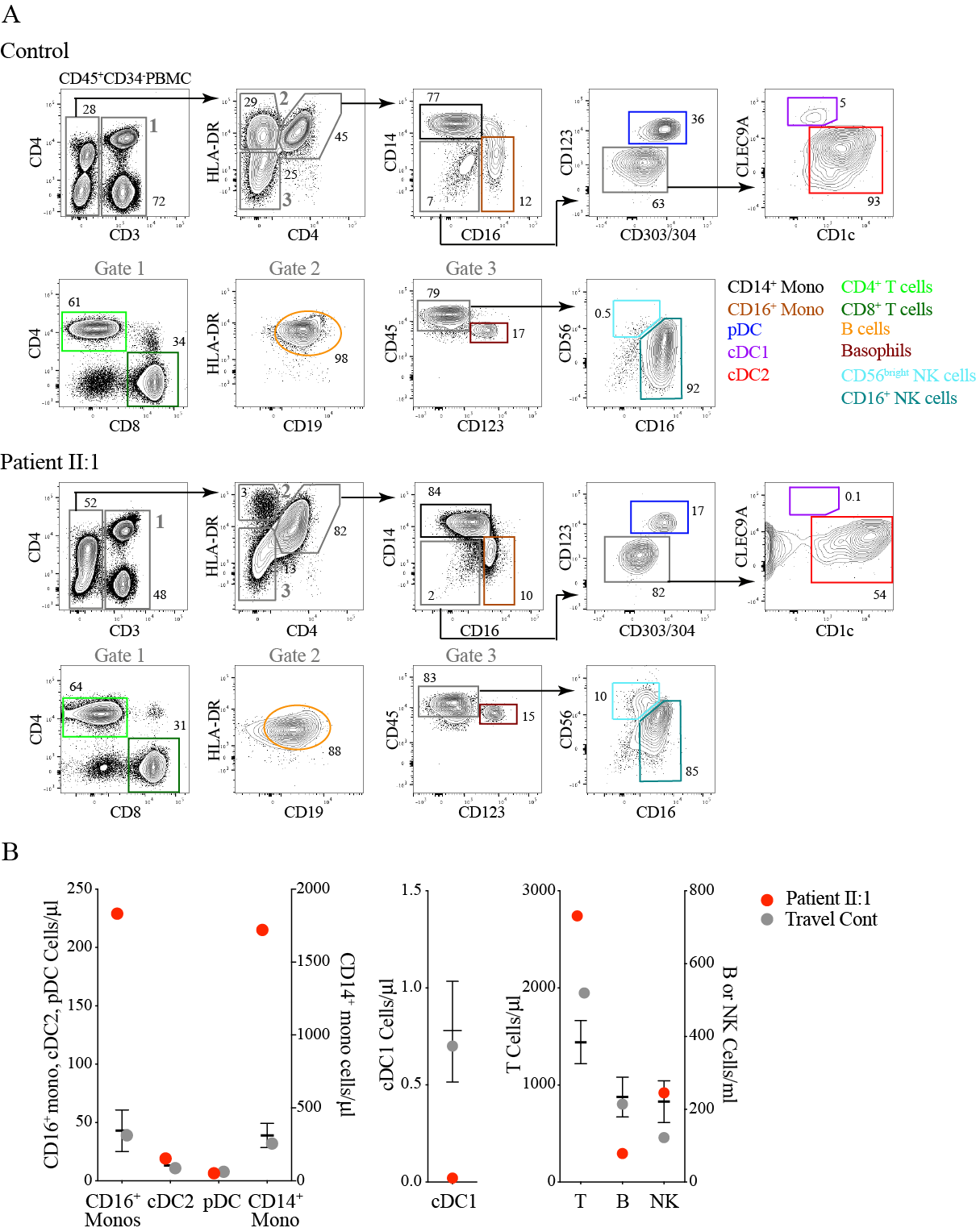
Figure E1.** Absolute cell counts from whole blood of the proband during acute illness (second admission) and a healthy control (data from a single experiment). (A). Flow cytometric analysis of Trucount™ cell quantification and phenotyping from whole blood identified CD4^+^ and CD8^+^ CD3^+^ T cells (Gate 1 and light or dark green gates, respectively), HLA-DR^+^CD4^-^CD19^+^ B cells (Gate 2 and orange gate), HLA-DR^-^CD4^-^CD123^+^CD45^low^ basophils (Gate 3 and burgundy gate), CD56^bright^ and CD16^+^ NK cells (Gate 3 and turquoise and teal gates, respectively), HLA-DR^+^CD4^+^ CD14^+^ monocytes (black gate), CD16^+^ monocytes (brown), CD123^+^CD303/4^+^ pDC, CLEC9A^+^ cDC1 (purple) and CD1c^+^ cDC2 (red). Gated populations quantified as percent of parent gate.

(B). Summary histograms show the absolute monocyte, dendritic cell and lymphocyte subset counts from the proband (red dots) and a healthy travel control (gray dots). Bars represent mean + 95% CI of n=18 healthy controls.


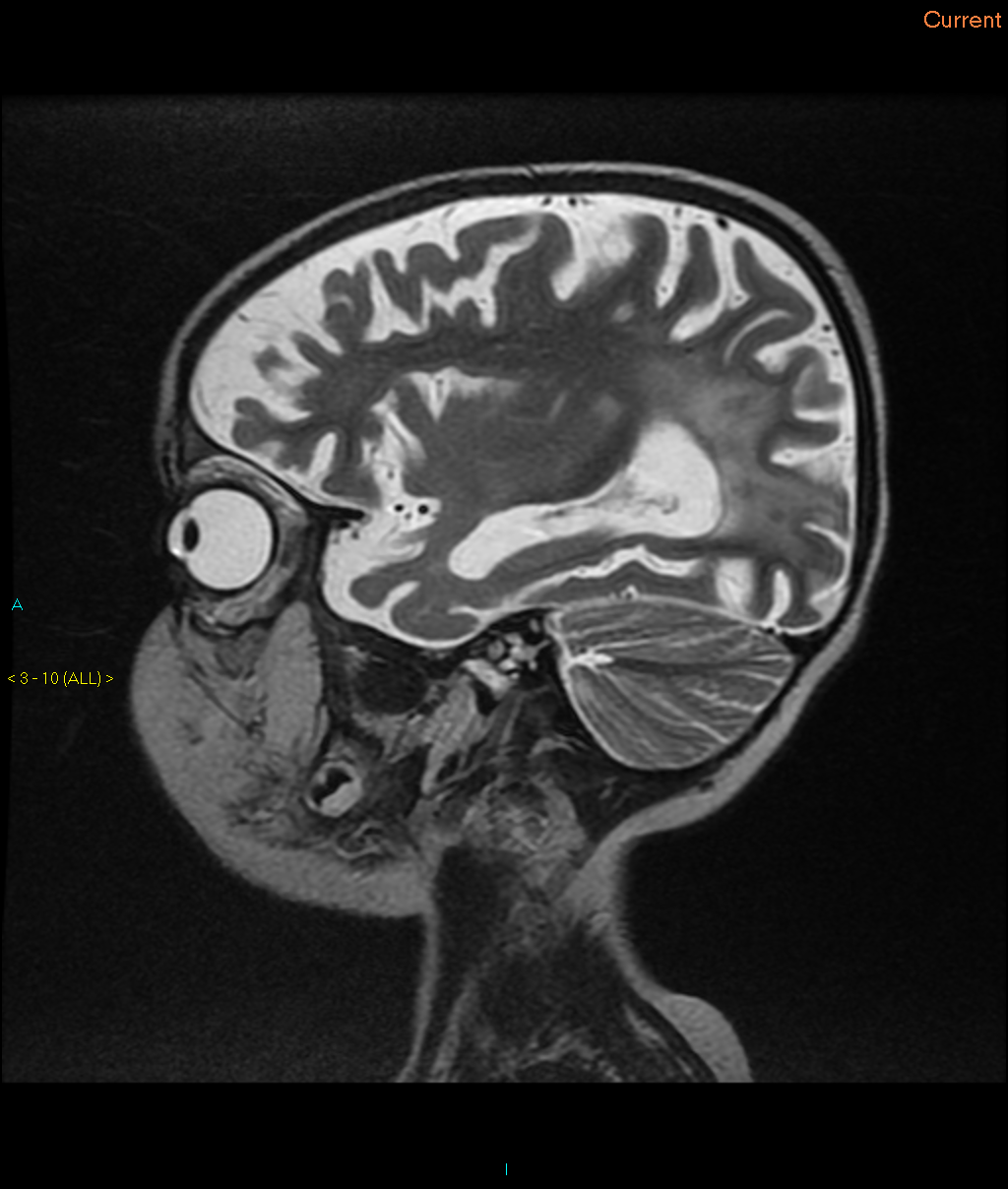

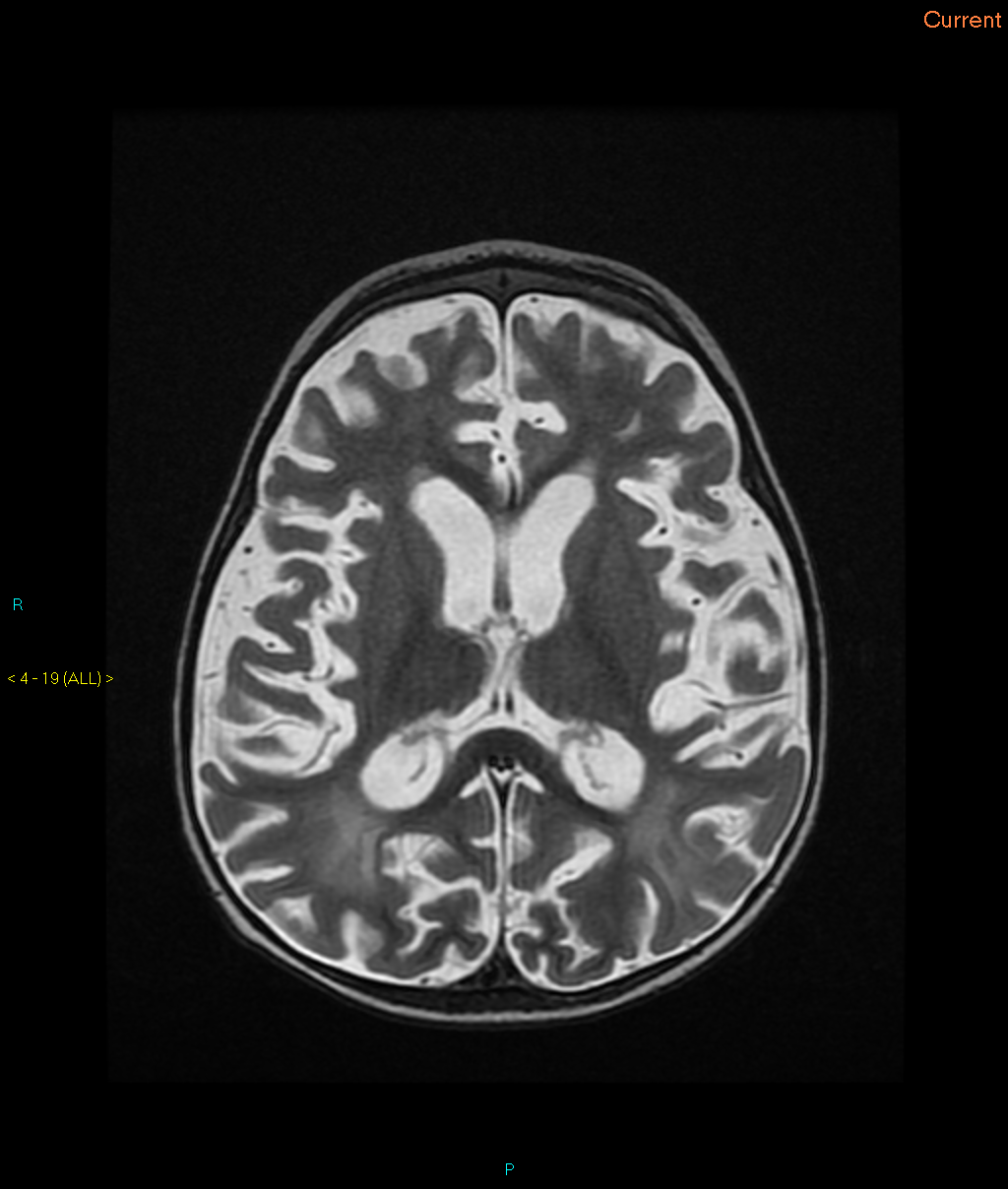

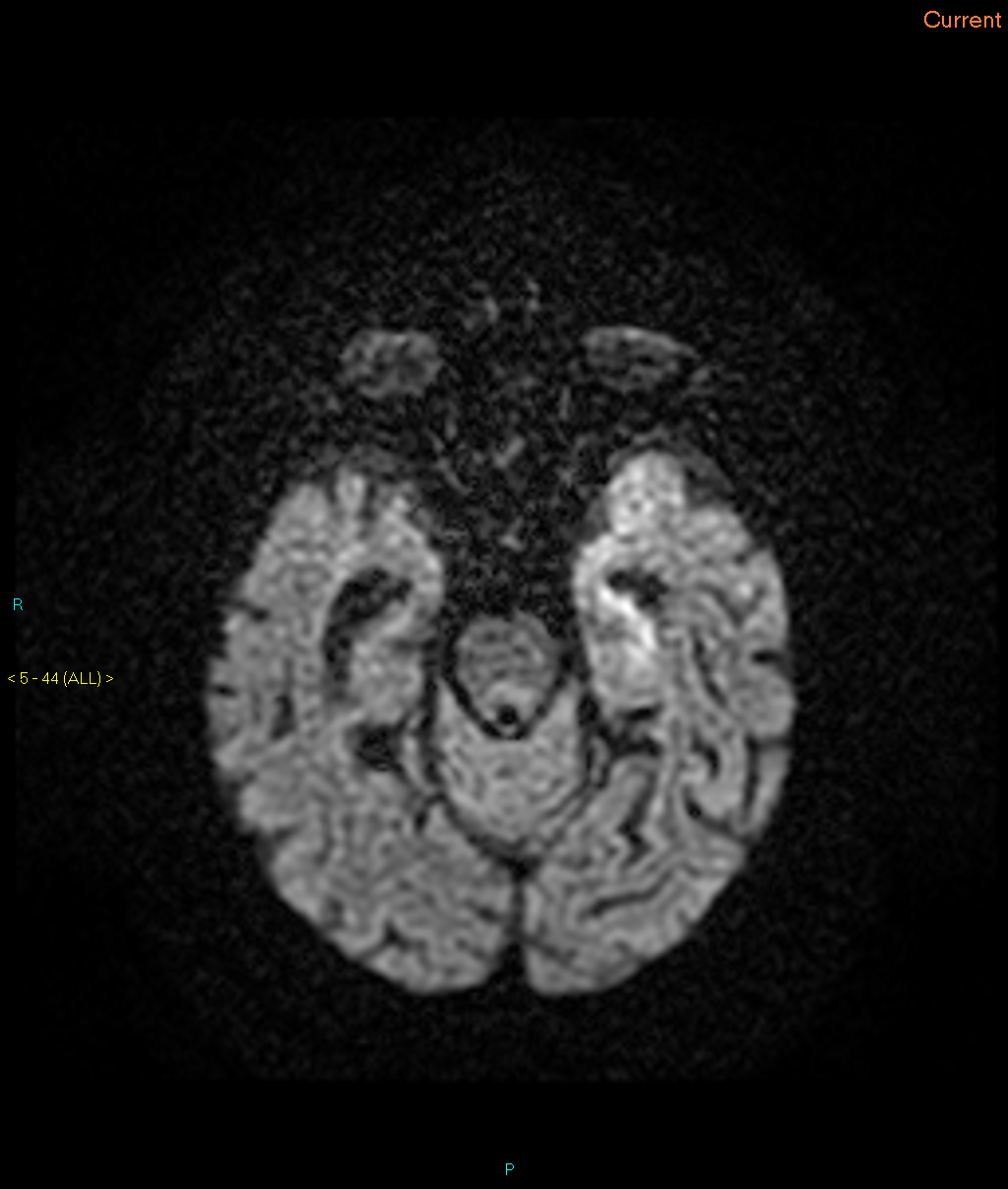

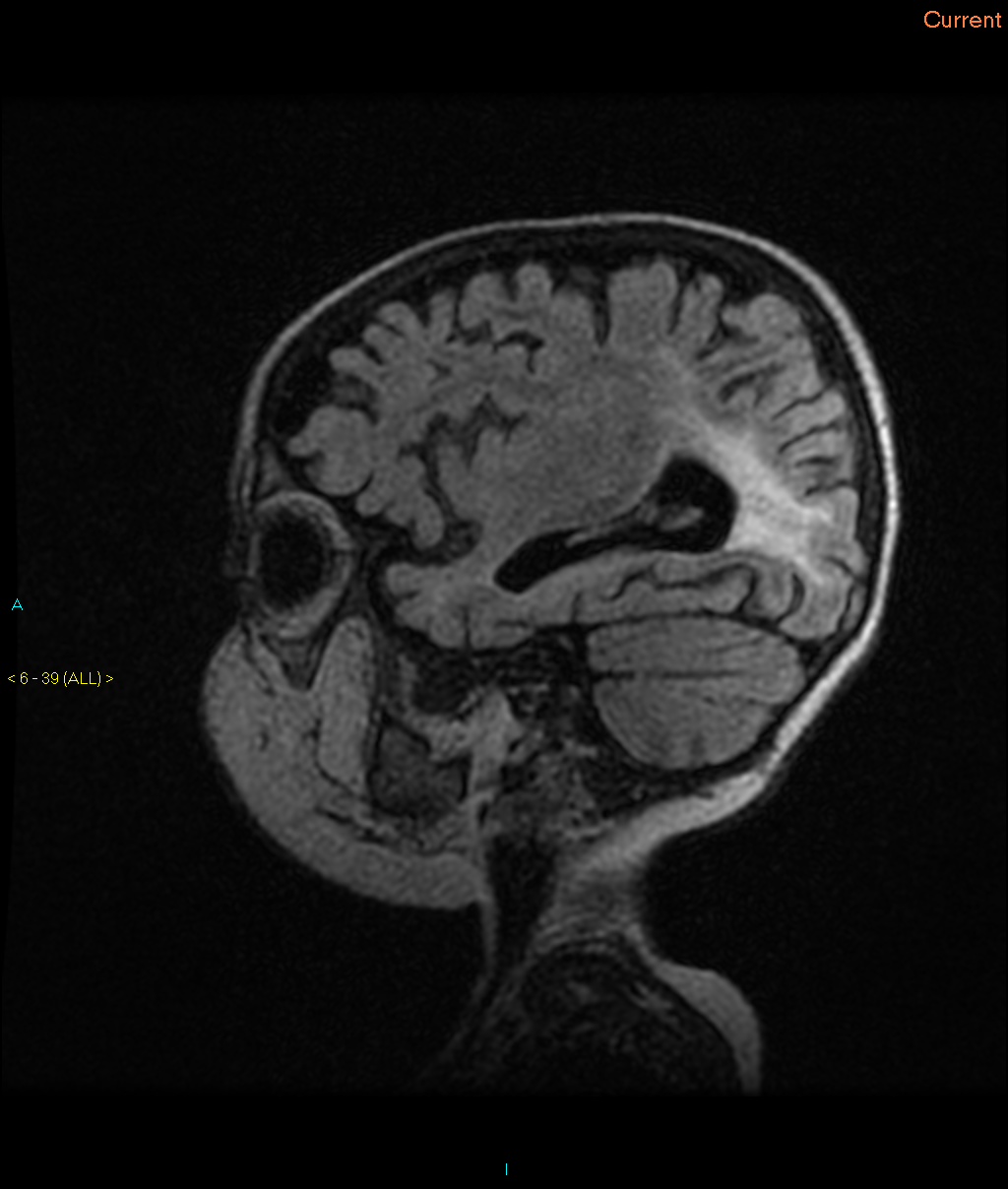

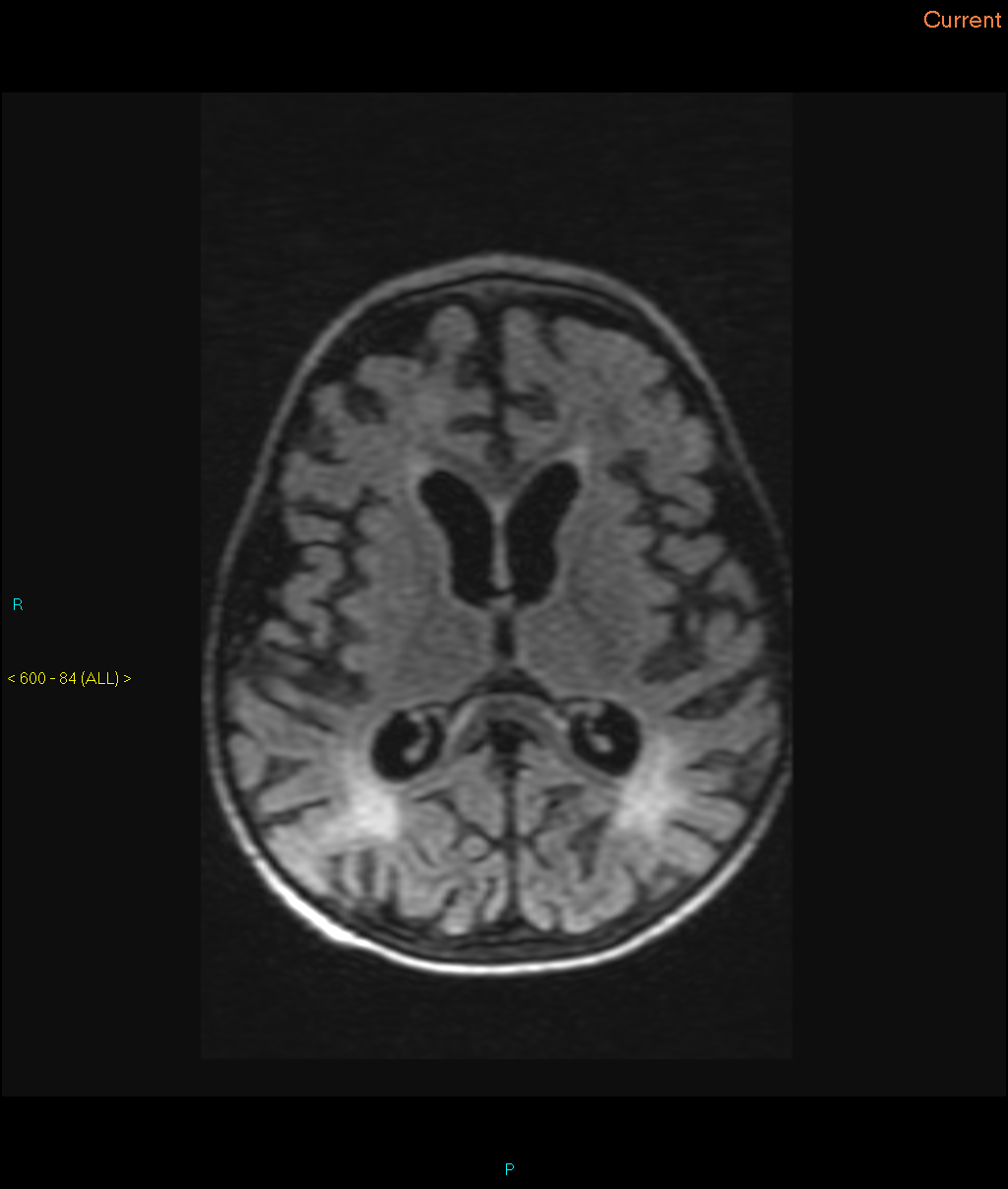


A

B

C

D

E

**Figure E2.** First magnetic resonance imaging performed on day 81. (A) sagittal T2, (B) axial T2, (C) sagittal FLAIR, (D) axial FLAIR, (E) axial DWI, demonstrating symmetric, bilateral white matter changes in the parieto-occipital region, diffusion restriction in the left hippocampus and generalized cerebral atrophy. FLAIR Fluid-attenuated inversion recovery, DWI Diffusion-weighted imaging.


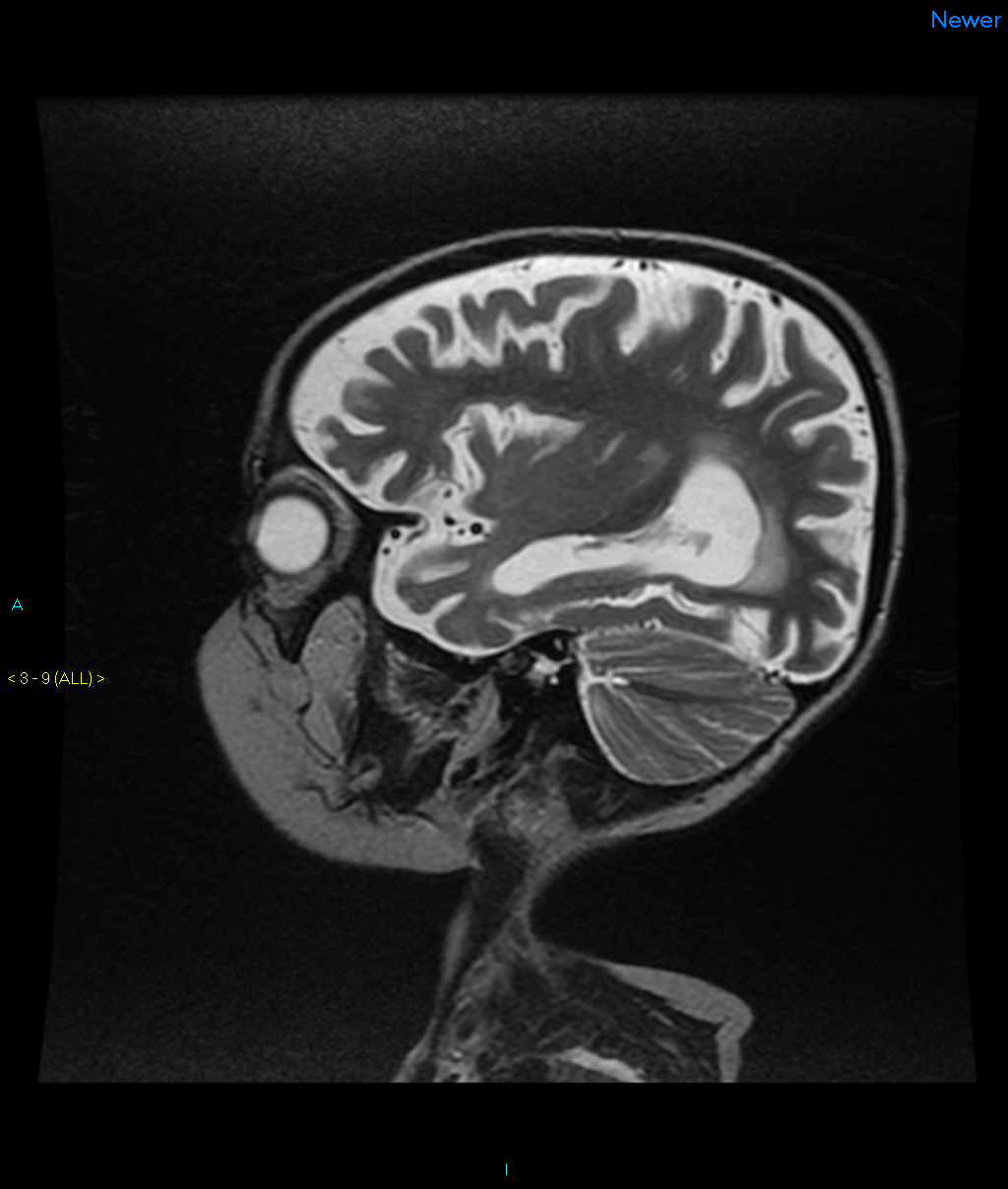

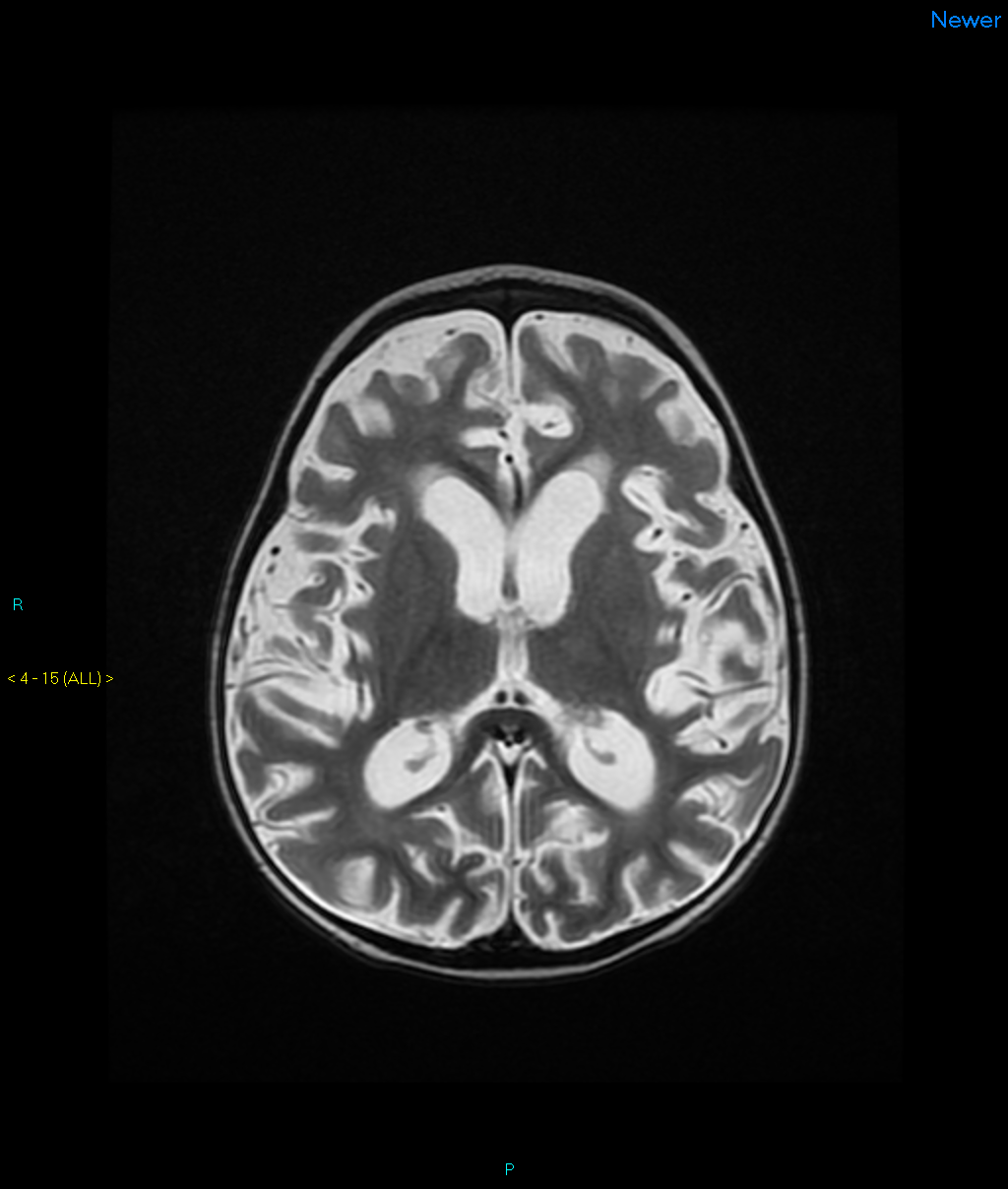

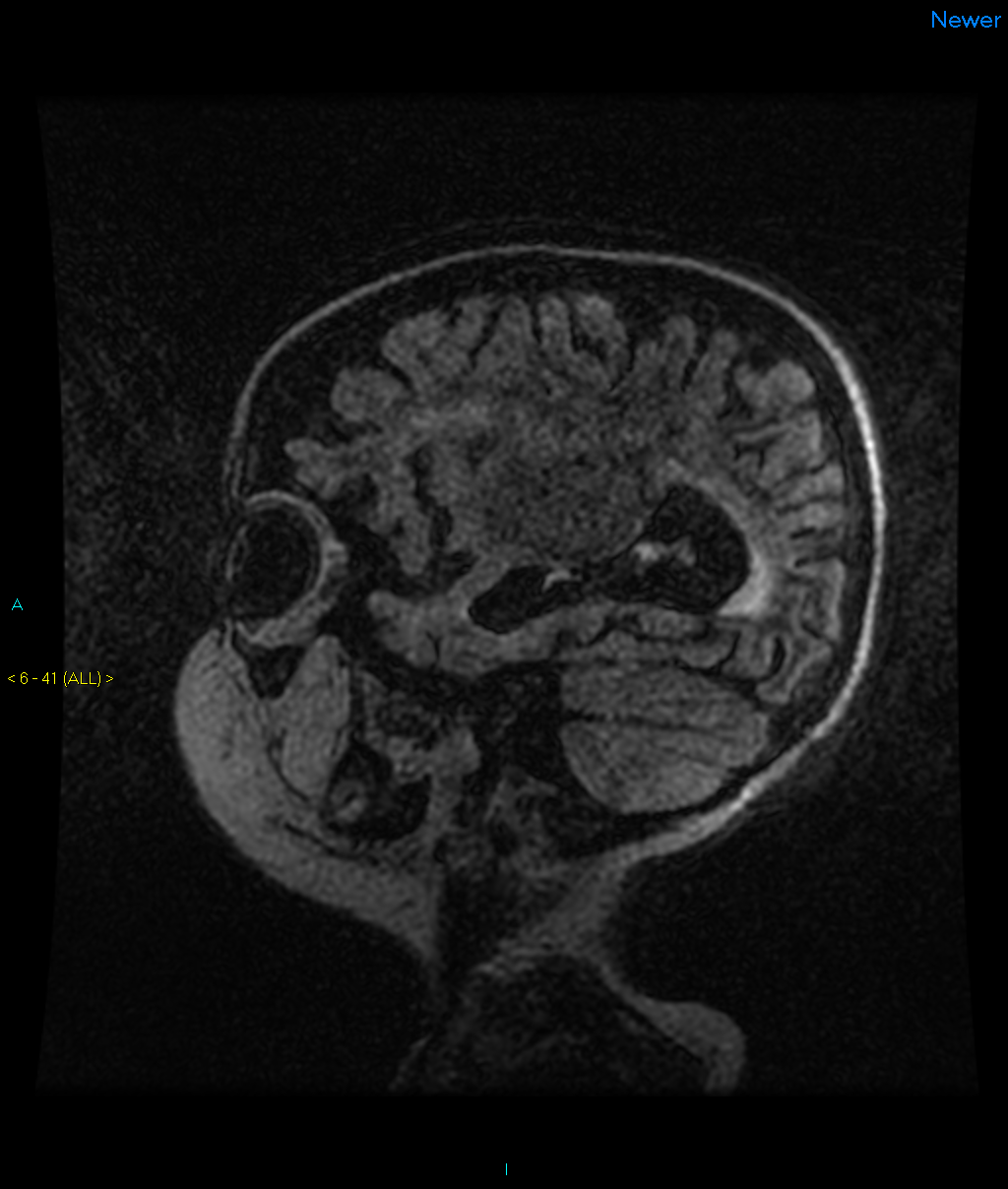

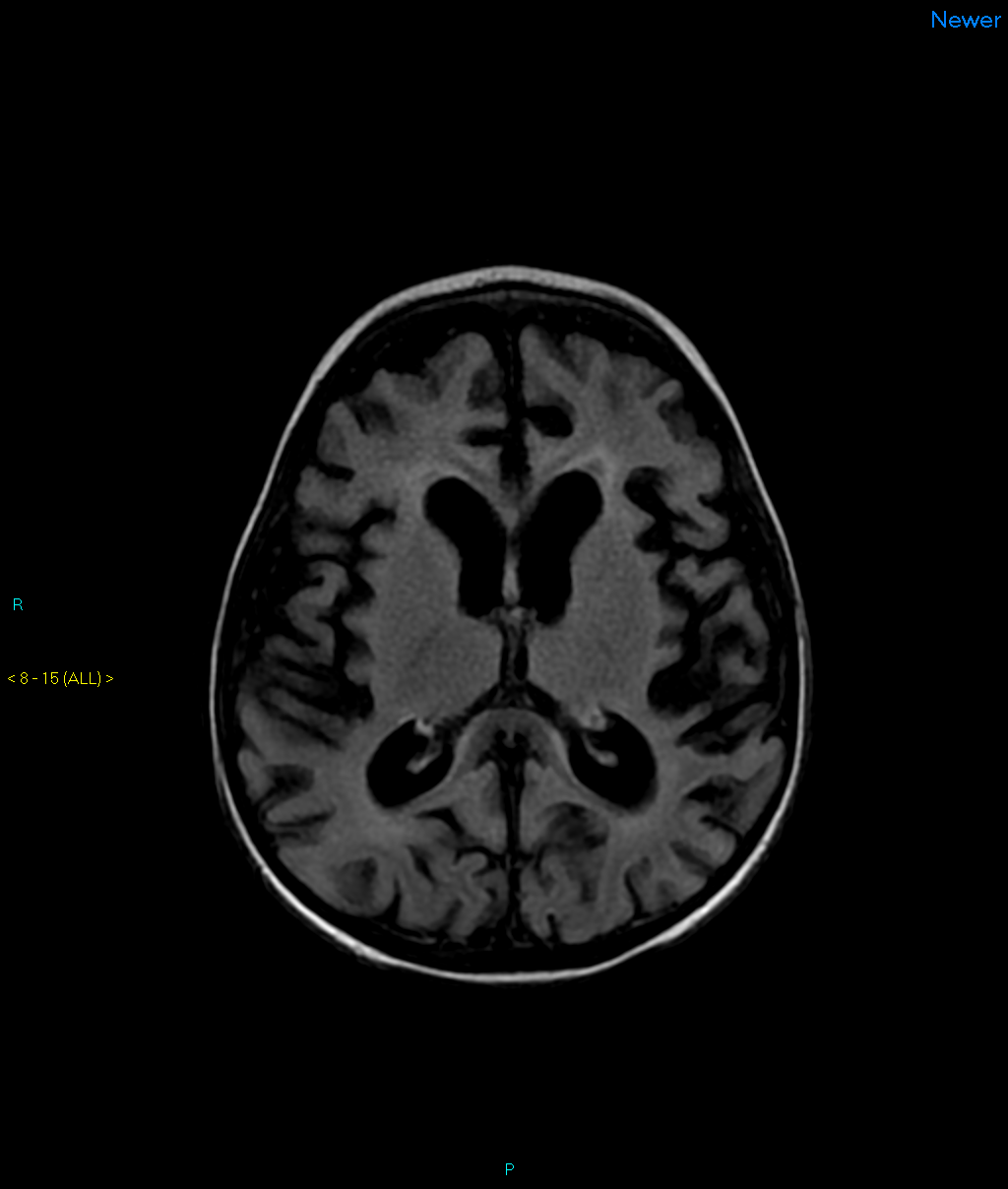


A

B

C

D

**Figure E3.** Follow-up magnetic resonance imaging performed on day 122. (A) sagittal T2, (B) axial T2, (C) sagittal FLAIR, (D) axial FLAIR, showing improvement in the white matter changes but worsened generalized cerebral atrophy. FLAIR Fluid-attenuated inversion recovery, DWI Diffusion-weighted imaging.

**Figure E4.** Course of the inflammatory markers CRP (black) and IL-6 (blue) over time alongside immunomodulatory treatment. Arrows indicate dates of lumbar punctures. CsA Ciclosporin A, IVIG intravenous immunoglobulins.


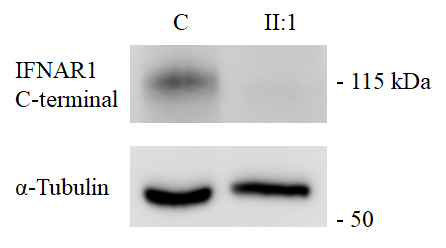


**Figure E5.** IFNAR1 Immunoblot using C-terminal antibody.


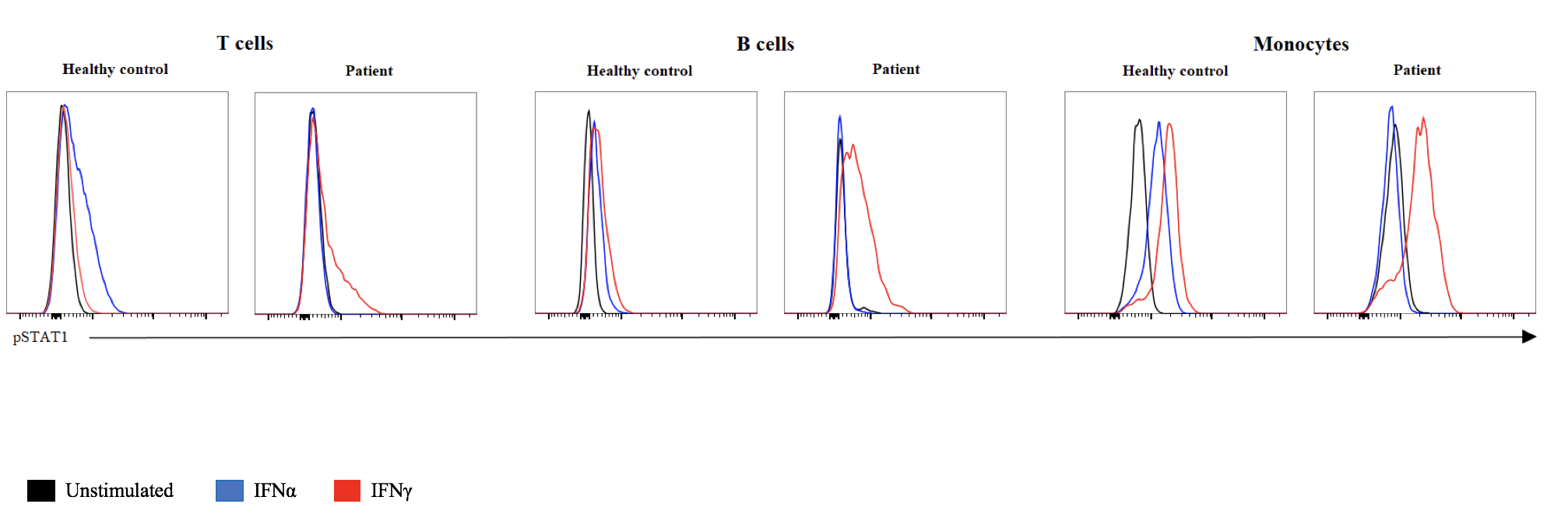


**Figure E6.** Phosflow analysis confirming lack of IFNAR response, as shown by pSTAT1 fluorescence intensity, in patient lymphocytes and monocytes in response to IFNs (data from a single experiment).

**
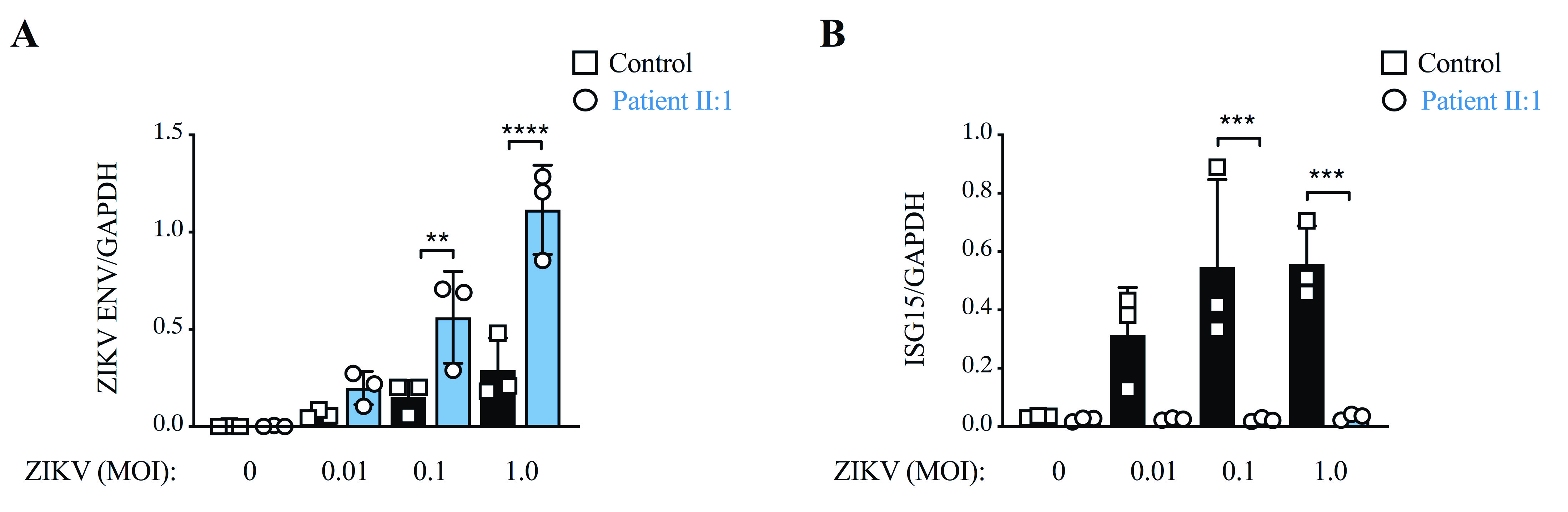
**

**Figure E7**. Densitometry analysis of immunoblots showing (A) ZIKV ENV and (B) ISG15. Mean ± SD ** P<0.01, ***P<0.01, ****P<0.001, Two-way ANVOA with Tukey’s post-test.

**Supplementary references**

1. Kralickova P, Milota T, Litzman J, Malkusova I, Jilek D, Petanova J, et al. CVID-Associated Tumors: Czech Nationwide Study Focused on Epidemiology, Immunology, and Genetic Background in a Cohort of Patients With CVID. Front Immunol. 2018;9:3135.

2. Siepel A, Bejerano G, Pedersen JS, Hinrichs AS, Hou M, Rosenbloom K, et al. Evolutionarily conserved elements in vertebrate, insect, worm, and yeast genomes. Genome Res. 2005;15(8):1034-50.

3. Schwarz JM, Rodelsperger C, Schuelke M, Seelow D. MutationTaster evaluates disease-causing potential of sequence alterations. Nat Methods. 2010;7(8):575-6.

4. Rentzsch P, Witten D, Cooper GM, Shendure J, Kircher M. CADD: predicting the deleteriousness of variants throughout the human genome. Nucleic acids research. 2019;47(D1):D886-D94.

5. Duncan CJA, Thompson BJ, Chen R, Rice GI, Gothe F, Young DF, et al. Severe type I interferonopathy and unrestrained interferon signaling due to a homozygous germline mutation in STAT2. Sci Immunol. 2019;4(42).
